# Ixazomib-associated tumor lysis syndrome in multiple myeloma: A case report

**DOI:** 10.1097/MD.0000000000022632

**Published:** 2020-11-06

**Authors:** Fengbo Jin, Mingzhen Yang, Yingying Chen, Lei Jiang, Lixia Liu

**Affiliations:** The Fourth Affiliated Hospital of Anhui Medical University, Hefei, China.

**Keywords:** ixazomib, kidney function, multiple myeloma, proteasome inhibitors, tumor lysis syndrome

## Abstract

**Rationale:**

Tumor lysis syndrome (TLS) is an oncologic emergency, but its incidence in MM is rare. To our knowledge, ixazomib has not been associated with TLS in MM.

**Patient concerns:**

The patient developed TLS after 10 days of treatment with ixazomib, accompanied by renal failure of hyperuricemia, hyperkalemia, and hyperphosphatemia.

**Diagnoses:**

MM (type IgG λ) was diagnosed according to the diagnostic criteria established by the International Myeloma Working Group and classified stage IIA by the International Staging System. TLS was diagnosed after the patient met all three criteria of the Cairo-Bishop TLS scoring system.

**Interventions:**

From April 8, 2017, the patient was treated with 3 courses of bortezomib, cyclophosphamide, and dexamethasone chemotherapy. From August 18, she received five courses of bortezomib combined with DCEP chemotherapy. On May 21, 2018 treatment was switched to lenalidomide, bortezomib, and dexamethasone for four courses. Ixazomib was started on October 10, 2018 with cyclophosphamide and dexamethasone. On October 19, 2018 vigorous intravenous hydration with sodium bicarbonate was initiated and peroral febuxostat was administered.

**Outcomes:**

On October 19, changes in hematological indicators raised concern for TLS worsening kidney function and decreasing urine output. She refused renal replacement treatment for TLS-induced acute kidney injury. On October 26th, the patient died of respiratory failure.

**Lessons:**

This case highlights the need to vigilant for the occurrence of TLS in patients undergoing MM treatment with ixazomib. Higher baseline uric acid or creatinine, rapidly progressive anemia, and raised lactate dehydrogenase (LDH) and β2-microglobulin may be surrogate markers of TLS.

## 1 Introduction

Tumor lysis syndrome (TLS) is characterized by a combination of metabolic disturbances resulting from the rapid release of neoplastic intracellular contents.^[1]^ This efflux of intracellular constituents into the extracellular space may damage normal homeostatic mechanisms, leading to TLS and life-threatening acute kidney injury, arrhythmias, and neurologic complications.^[2]^ It is an oncologic emergency either due to administration of cytotoxin in the treatment of malignancy or due to the progression of an aggressive disease. Plasma cells in multiple myeloma (MM) proliferate slowly with only a small proportion in S phase at any given time.^[3]^ Therefore, TLS is rare in MM. Ixazomib is a second-generation proteasome inhibitor. Like bortezomib and carfilzomib, ixazomib primarily inhibits the chymotrypsin-like (β5) proteolytic site within the 20S proteasome.^[4]^ The inhibition of this enzyme results in the accumulation of excess proteins, leading to cell cycle arrest and apoptosis.^[4]^ Ixazomib is used in a growing number of patients with relapsed MM,^[5]^ but to the best of our knowledge, no relevant literature focused on ixazomib-induced TLS. We present a case of ixazomib-induced TLS in MM and the related literature review.

## 2 Case presentation

Standard care was performed, so ethical approval was not applicable in this study. Written informed consent was obtained from the patient.

A 63-year-old woman suffered from back pain for one year that progressively worsened and became intolerable. She was admitted to the outpatient department in March 2017. Physical examination showed consciousness, good general mental state, no palpable superficial lymph nodes, no tenderness of the sternum, clear breath sounds in both lungs, no rales, heart rate at 64 bpm, regular rhythm, no cardiac souffle was heard, soft abdomen, no tenderness of the abdomen, and no swelling of the lower limbs. Routine blood tests revealed white blood cells at 3.72 × 10^9^/L, platelets at 182 × 10^9^/L, hemoglobin (Hb) at 111 g/dL, calcium at 2.01 mmol/L, creatinine at 76 μmol/L, albumin at 37.8 g/l, and β2 microglobulin at 3.7 mg/L. More in-depth investigation showed IgGλ paraprotein at 23.8 g/L. Urine was positive for Bence Jones proteins (0.53 g/L). Bone marrow biopsy revealed 17% plasma cell infiltration, with del(13q14) and del(17p13) detected by fluorescence in situ hybridization (FISH). Multiple myeloma (type IgG λ) was diagnosed according to the MM diagnostic criteria established by the International Myeloma Working Group (IMWG),^[6]^ and according to the International Staging System (ISS) was stage IIA.^[7]^ According to the 2017 National Comprehensive Cancer Network (NCCN) guidelines,^[8]^ on April 8, 2017, the patient was treated with bortezomib (1.3 mg/m^2^, days 1, 4, 8, and 11), cyclophosphamide (300 mg/m^2^, days 1, 8, 15, and 22), and dexamethasone (20 mg/d, days 1-2, 4-5, 8-9, and 11-12). The second course started on May 31, 2017, and the third course started on July 13, 2017. IgG paraprotein decreased to 11.9 g/L after three courses of chemotherapy. After the three courses of treatment, the disease condition was evaluated. IgG was reduced to 11.9 g/L. Routine blood analysis showed white blood cells at 4.6 × 10^9^/L, hemoglobin at 121 g/L, platelets at 140 × 10^9^/L, liver function and renal electrolytes were normal, bone marrow cytology showed no abnormal plasma cells, and 0.1% plasma cells were detected by flow cytometry. The evaluation results were partial remission.

From August 18, 2017 to March 6, 2018, 5 courses of bortezomib combined with DCEP chemotherapy were administrated (Bortezomib 1.3 mg/m^2^, d1,8; cisplatin 10 mg/m^2^/d, d1-4; cyclophosphamide 400 mg/m^2^/d, d1-4; etoposide 40 mg/m^2^/d, d1-4; dexamethasone 40 mg/d, d1-4). In December 2017, the disease condition was still partially relieved, and the routine blood analysis and liver and kidney functions were normal.

In May 2018, the patient suffered from progressive anemia (Hb of 84 g/L) and pain at the waist. Liver function showed globulin at 39.2 g/L, while the remaining indicators were in the normal range. IgG was at 26.02 g/L, creatinine at 43.6 μmol/L, and β2-MG at 8.9 g/L. Magnetic resonance imaging (MRI) showed multiple lytic lesions in the lumbar spine. Reexamination of bone marrow cytology detected 5% plasma cells, and flow cytometry detected 4% abnormal plasma cells. This was considered as disease progression.

Treatment was switched to lenalidomide (25 mg/d, 21-day cycles), weekly bortezomib (1.3 mg/m^2^), and dexamethasone for four courses (the courses started on May 21, 2018, June 23, 2018, July 2, 2018, and August 21, 2018). The best achieved response was a drop in IgG paraprotein to 11.6 g/L after the third course. This later rose to 52.65 g/L after the fourth course, and with a drop in Hb to 64 g/l, with lactate dehydrogenase (LDH) at 187 U/L and β2-microglobulin at 13.7 mg/L. The bone marrow showed no change in plasma cell burden, with 20% plasma cell infiltrate. The liver and kidney function levels were in the normal range during this period.

Ixazomib was started in October 2018. The specific regimens were ixazomib 4 mg/d, d1, d8, d15; cyclophosphamide 300 mg/m^2^/d, d1, d8, d15, d22; and dexamethasone 20 mg/d, d1-2, d8-9, d15-16. The patient's body functions normalized after the first dose of cycle 1 on October 10, 2018. After the second dose of ixazomib, the patient developed repeated chest tightness and shortness of breath, and progressive exacerbation. On October 21, arterial blood gas showed an oxygenation index of 550. On October 19, creatinine rose from 90.3 to 216.1 μmol/L, while potassium rose from 3.40 to 4.75 mmol/L. Uric acid was at 1240 μmol/L, urea at 27.10 mmol/L, and LDH at 594 IU/L. The changes in hematology indicators are shown in Table 1.

**Table 1:** Serum parameters before and after ixazomib usage.

Serum Parameter (units)	October 8 (Before Ixazomib Usage)	October 19 (After Ixazomib Usage)	October 25 (After Ixazomib Usage)
Sodium (mmol/L)	139.6	140.9	135.3
Potassium (mmol/L)	3,85	4.73	1.95
Chloride (mmol/L)	111.8	114.2	103.1
Urea nitrogen (mmol/L)	6.01	27.1	41.6
Creatinine (μmol/L)	76.4	216.1	391.7
Glucose (mmol/L)	5.86	7.19	6.7
Calcium (mmol/L)	2.46	2.23	1.5
Protein (g/L)	95.9	73.3	61.2
Albumin (g/L)	27.4	19.3	23.8
AST (IU/L)	25	159	45
ALT (IU/L)	20	75	45
Alkaline phosphatase (IU/L)	55	52	41
Total bilirubin (μmol/L)	8.9	3.9	5.6
Phosphorus (mmol/L)	1.45	3.84	1.95
Uric acid (μmol/L)	395	1240	534
Lactate dehydrogenase (IU/L)	187	594	342
White blood cells (K/μl)	2.99	3.51	0.53
Hemoglobin (g/dl)	7.8	53	56
Platelets (K/μl)	56	42	14

These laboratory findings raised concern for TLS. Vigorous intravenous hydration with sodium bicarbonate was initiated. On October 19th, peroral febuxostat was administered. On October 25th, uric acid was reduced to 534 μmol/l, but with worsening kidney function and decreasing urine output. She refused renal replacement treatment for TLS-induced acute kidney injury (AKI). Eventually, on October 26th, the patient died of respiratory failure.

## 3 Discussion and conclusions

We present the case of a 63-year-old female with MM and severe hyperuricemia. The patient's potassium level increased by 25% from baseline. Hyperphosphatemia and acute renal failure both occurred within 10 days after starting ixazomib therapy for MM. To the best of our knowledge, this is the first reported case of TLS associated with ixazomib treatment in MM. There are many reports of bortezomib-induced tumor lysis,^[9,10]^ but no case of ixazomib-related TLS has been reported. This patient had normal liver, kidney function, and electrolytes during treatment with bortezomib. There were no corresponding manifestations of TLS before administration of ixazomib. After oral administration of the second dose of ixazomib, the patient's creatinine levels increased, uric acid elevated, and electrolytes were disturbed. This led to a diagnosis of combined tumor lysis. Therefore, it was considered to be ixazomib-induced TLS.

Ixazomib is a peptide boronic acid proteasome inhibitor, which is approved for the treatment of relapsed, refractory, or relapsed and refractory MM. In the recent phase 3 clinical trial TOURMALINE-MM1, there was no occurrence or mention of TLS in patients treated with ixazomib.^[11]^ Here, our patient met all three criteria (i.e., hyperuricemia, hyperphosphatemia, and 25% increase from baseline for potassium) of the Cairo-Bishop TLS scoring system,^[12]^ and only after 10 days of ixazomib treatment. Moreover, the patient had an elevation of creatinine by >1.5 times the upper limit of normal, thereby meeting the criteria of clinical TLS.^[12]^

TLS is a relatively rare complication of MM. The incidence of TLS associated with MM has seldom been evaluated in large-scale studies after TLS guidelines were published.^[12]^ In a retrospective single-center case series, the prevalence of TLS in MM was 10.5%, and the presence of circulating plasma cells, higher ISS score for MM, higher baseline uric acid, and creatinine were all risk factors for the development of TLS.^[9]^ Suzuki et al^[10]^ investigated the parameters that could predict the development of TLS in patients undergoing bortezomib treatment. They retrospectively reviewed 35 patients with relapsed or refractory myeloma treated with bortezomib-containing regimens; TLS occurred in six patients (17.1%) during the first course of bortezomib-containing treatment. They also found that TLS occurred more often in patients with rapidly progressive anemia.^[10]^ Nevertheless, similar characteristics to those denoting higher likelihood of developing TLS in the pre-bortezomib era were found in a group of at-risk patients, heavy plasma cell burden, rapidly progressive disease, and cytogenetic aberrations (chromosome 13 in particular). The TLS incidence in that group of at-risk patients was 1.10% (9/820).^[9,10]^ Officially, bortezomib is considered to be uncommonly associated with TLS. In a study of 17 patients with relapsed myeloma, three developed TLS, all three patients having significant disease burden and elevated LDH and β2-microglobulin levels.^[9,10]^ TLS was also reported to be found in patients who were treated with thalidomide,^[13]^ carfizomib,^[14]^ and elotuzumab.^[15]^

The case reported here had many of the risk factors for TLS development. Indeed, she had refractory disease having failed to respond to multiple previous therapies and high β2-microglobulin levels. In addition, TLS developed within the first cycle of treatment with ixazomib. She demonstrated adverse karyotypic features and rapidly progressive anemia.

Over recent years, TLS has appeared to occur more frequently in patients with myeloma, possibly because patients are treated more aggressively with new agents. As more active agents are being introduced into the therapeutic armamentarium for MM, higher awareness is needed regarding the potential of the development of TLS in patients with myeloma. Higher baseline uric acid or creatinine, rapidly progressive anemia, and raised LDH and β2-microglobulin may be surrogate markers of TLS development. The merit of cytogenetic analysis of TLS is as yet unclear, with a possibility of a deletion in chromosome 13.

In conclusion, with the increasing use of new regimens in the treatment of aggressive MM, TLS may occur more frequently. The case reported here showed ixazomib-associated TLS, which presented with obvious hyperuricemia, hyperkalemia, hyperphosphatemia, and acute renal failure within 10 days after starting ixazomib therapy for MM. It is important to be aware of the patients with surrogate risk factors for TLS, in order to give appropriately targeted prophylaxis.

## Author contributions

**Case discussion and interpretation:** Yingying Chen, Lei Jinang, Lixia Liu.

**Conceptualization:** Fengbo Jin, Mingzhen Yang.

**Final approval of manuscript:** Fengbo Jin, Mingzhen Yang, Yingying Chen, Lei Jiang, Lixia Liu.

**Revision of manuscript:** Fengbo Jin.

**Writing manuscript:** Fengbo Jin.
